# Cave-Associated Histoplasmosis Outbreak Among Travelers Returning from Costa Rica — Georgia, Texas, and Washington, December 2024–January 2025

**DOI:** 10.15585/mmwr.mm7417a1

**Published:** 2025-05-15

**Authors:** Ria R. Ghai, Elizabeth T. Sajewski, Mitchell Blass, Hayley Belles, Hope Dishman, Julie Gabel, BreeAnna Dell, Mariah Harper, Hanna N. Oltean, Olivia Smith, Emeka Ogwuegbu, Saad Zaheer, Alexander Jordan, Meghan Lyman, Ian Hennessee, Mitsuru Toda

**Affiliations:** ^1^Division of Foodborne, Waterborne, and Environmental Diseases, National Center for Emerging and Zoonotic Diseases, CDC; ^2^Epidemic Intelligence Service, CDC; ^3^St. Joseph’s Hospital, Atlanta, Georgia; ^4^Georgia Department of Public Health; ^5^Public Health – Seattle & King County, Seattle, Washington; ^6^Washington State Department of Health, Tumwater, Washington; ^7^Texas Department of State Health Services; ^8^Dallas County Health and Human Services, Dallas, Texas.

**Summary**

**What is already known about this topic?**

Histoplasmosis is a fungal infection that primarily affects the lungs; it is caused by *Histoplasma* organisms, which are often found in soil contaminated with bird or bat droppings. The condition is often misdiagnosed.

**What is added by this report?**

CDC was notified about a cluster of suspected histoplasmosis cases affecting 12 members of an extended family from three U.S. states. They had all visited Venado Caves in Costa Rica, the site of a previous histoplasmosis outbreak associated with contact with bat droppings, the likely source of exposure. Four family members received fungal antigen testing, and two received test results positive for *Histoplasma capsulatum* species complex.

**What are the implications for public health practice?**

Health care providers should consider a diagnosis of histoplasmosis in patients who have signs and symptoms compatible with the condition, including fever, malaise, cough, headache, chest pain, chills, and myalgias. Obtaining a detailed travel and activity history is necessary to identify exposure to bat or bird droppings in caves or elsewhere.

## Introduction

Histoplasmosis is a fungal infection that primarily affects the lungs. The condition is caused by *Histoplasma* organisms*,* which are often found in soil contaminated with bird or bat droppings. On January 17, 2025, a Georgia infectious disease physician notified CDC of suspected histoplasmosis cases among 12 members of an extended family from households in Georgia, Texas, and Washington. The ill family members included six adults aged 42–49 years and six children aged 8–16 years. They had recently returned from Costa Rica, where they toured a cave linked to a previous histoplasmosis outbreak (*1*).

## Investigation and Outcomes

On January 21, CDC launched a multistate investigation in collaboration with the Georgia Department of Public Health, Texas Department of State Health Services, and Washington State Department of Health to identify and characterize cases. This activity was reviewed by CDC, deemed not research, and was conducted consistent with applicable federal law and CDC policy.*

Thirteen family members traveled to Arenal and Manuel Antonio, Costa Rica, during December 21–28, 2024. Investigation revealed that 12 of the 13 family members toured Venado Caves, a popular tourist location, on December 24 (day 0). All reported seeing bats and having direct contact with bat droppings while crawling and squeezing through tight spaces in the cave. All 12 persons became mildly or moderately ill after returning to the United States, 8–19 days after exposure. Signs and symptoms included headache, malaise, fever, night sweats, myalgias, and respiratory and gastrointestinal symptoms (Table). The one family member who did not tour the cave did not become ill. No other activities reported before, during, or after the trip were associated with known risk for exposure to *Histoplasma* species.

**TABLE:** Demographic and clinical characteristics of patients with confirmed, probable, or suspected cave-associated histoplasmosis after travel to Costa Rica — Georgia, Texas, and Washington, 2024

Characteristic	Cases,* no. (%)			
	Confirmed (n = 1)	Probable (n = 8)	Suspected (n = 3)	Total (N = 12)
**Age group, yrs**				
<10	0 (—)	0 (—)	1 (33)	**1 (8)**
11–17	0 (—)	3 (38)	2 (67)	**5 (42)**
18–39	0 (—)	0 (—)	0 (—)	**0 (—)**
41–64	1 (100)	5 (63)	0 (—)	**6 (50)**
≥65	0 (—)	0 (—)	0 (—)	**0 (—)**
**Sex**				
Female	0 (—)	5 (63)	2 (66)	**7 (58)**
Male	1 (100)	3 (38)	1 (33)	**5 (42)**
**State of residence**				
Georgia	1 (100)	2 (25)	1 (33)	**4 (33)**
Texas	0 (—)	3 (38)	1 (33)	**4 (33)**
Washington	0 (—)	3 (38)	1 (33)	**4 (33)**
**Antigen or antibody test results**				
Positive	1 (100)	1 (13)	0 (—)	**2 (17)**
Negative	0 (—)	2 (25)	0 (—)	**2 (17)**
No test performed	0 (—)	5 (63)	3 (100)	**8 (67)**
**Clinical course and treatment**				
Sought medical care	1 (100)	5 (63)	0 (—)	**6 (50)**
Hospitalized^†^	1 (100)	0 (—)	0 (—)	**1 (8)**
Received antifungal treatment	1 (100)	0 (—)	0 (—)	**1 (8)**
**Signs and symptoms**				
Extreme tiredness	1 (100)	8 (100)	0 (—)	**9 (75)**
Headache	1 (100)	6 (75)	2 (67)	**9 (75)**
Fever	1 (100)	7 (88)	0 (—)	**8 (67)**
Night sweats	1 (100)	5 (63)	0 (—)	**6 (50)**
Body aches	1 (100)	3 (38)	0 (—)	**4 (33)**
Cough	1 (100)	3 (38)	0 (—)	**4 (33)**
Chills	0 (—)	3 (38)	0 (—)	**3 (25)**
Difficulty breathing	1 (100)	1 (13)	1 (33)	**3 (25)**
Ear infection	1 (100)	1 (13)	0 (—)	**2 (17)**
Chest pain	1 (100)	1 (13)	0 (—)	**2 (17)**
Diarrhea	0 (—)	2 (25)	0 (—)	**2 (17)**
Hoarseness	1 (100)	1 (13)	0 (—)	**2 (17)**
Increased heart rate	0 (—)	2 (25)	0 (—)	**2 (17)**
Loss of appetite	0 (—)	2 (25)	0 (—)	**2 (17)**
Sore throat	0 (—)	2 (25)	0 (—)	**2 (17)**
Runny nose	0 (—)	1 (13)	0 (—)	**1 (8)**
Stomach pain	0 (—)	1 (13)	0 (—)	**1 (8)**
Vomiting	0 (—)	1 (13)	0 (—)	**1 (8)**

* The Council of State and Territorial Epidemiologists case definition for histoplasmosis includes both laboratory and clinical criteria; cases can be classified as confirmed or probable. One patient met the criteria for a confirmed case because the illness was clinically compatible (had at least two of the following: fever, chest pain, cough, myalgia, shortness of breath, headache, or erythema nodosum/erythema multiforme rash) and laboratory confirmed (fourfold or greater increase rise in *Histoplasma capsulatum* serum complement fixation antibody titers taken at least 2 weeks apart). Eight patients met the criteria for having a case of probable histoplasmosis, even though the condition was not laboratory confirmed, because their illness was clinically compatible, and they were epidemiologically linked to the patient with the confirmed case. In addition, three patients were suspected to have cases because although they each had only one clinically compatible finding (one with shortness of breath and two with headache), they were epidemiologically linked to the patient with the confirmed case.

^†^ The hospitalized patient had abnormal chest radiography findings that initially raised concerns about lung cancer. However, the travel history obtained the next day, which included touring a Costa Rican cave with bats, led to a suspected diagnosis of histoplasmosis. The patient received antifungal treatment and fully recovered.

Five adults and one child sought medical care at an urgent or a primary care facility; no travel history was elicited during their examinations. Among these six patients, three received antibiotics, two received corticosteroids, and one received a cough suppressant. One adult was referred to an emergency department and hospitalized because of abnormal chest radiography findings that raised concerns about lung cancer. However, when health care providers requested the patient’s travel history the next day and learned of the visit to a Costa Rican cave with bats, histoplasmosis became the suspected diagnosis. The patient initially was prescribed voriconazole and discharged 2 days after admission; treatment was subsequently changed to itraconazole (*2*,*3*). By 28 days after exposure, all 12 affected family members had recovered or were improving, consistent with the clinical course of mild or moderate histoplasmosis.

Fungal antigen testing was performed for four family members. Two received antigen test results positive for *Histoplasma capsulatum* species complex, including the hospitalized patient; test results for the other two patients were negative. Antigen testing for all four patients occurred within 1 month of symptom onset, the optimal time frame. However, because antigen detection sensitivity for histoplasmosis is lower in patients with mild disease who are immunocompetent, the negative test results might have been false negatives (*4*). One patient with a positive test result also received positive complement fixation antibody test results on two separate occasions. Also, in addition to the patient with chest radiography findings that initially raised concerns about lung cancer, another patient also had abnormal chest radiography results, including pulmonary nodules and opacities, consistent with histoplasmosis.

The 12 ill family members were classified as having confirmed (one patient), probable (eight patients), or suspected (three patients) histoplasmosis based on the Council of State and Territorial Epidemiologists (CSTE) case definition (*5*). The CSTE case definition includes both laboratory and clinical criteria; cases can be classified as confirmed or probable. Fungal antigen tests are considered nonconfirmatory laboratory criteria based on the CSTE case definition due to cross-reactivity with other fungal pathogens (*5*). One patient met the criteria for a confirmed case because the illness was clinically compatible (i.e., had at least two of the following: fever, chest pain, cough, myalgia, shortness of breath, headache, or erythema nodosum/erythema multiforme rash) and laboratory confirmed (fourfold or greater increase rise in *Histoplasma capsulatum* serum complement fixation antibody titers taken at least 2 weeks apart). Eight patients met the criteria for having a case of probable histoplasmosis because their illness was clinically compatible, and they were epidemiologically linked to the patient with the confirmed case, even though the condition was not laboratory confirmed. In addition, three patients were suspected to have cases because although they each had only one clinically compatible finding (one with shortness of breath and two with headache), they were epidemiologically linked to the patient with the confirmed case.

## Preliminary Conclusions and Actions

The same bat-inhabited cave in Costa Rica associated with this 2024 histoplasmosis outbreak was linked to a 1998–1999 outbreak among 51 persons (*1*), when *H. capsulatum* was isolated from bat droppings collected from the cave. In response to the outbreak described in this report, CDC alerted health departments through an Epidemic Information Exchange (Epi-X) notification to identify additional cases. CDC has been collaborating with the U.S. Embassy in Costa Rica and the Costa Rica Ministry of Health to incorporate information about histoplasmosis risks at this location into the caving tour waiver forms. In addition, the U.S. Embassy in Costa Rica issued a health alert in March 2025 notifying the public of the risk for contracting histoplasmosis from caving (*6*). Persons who have already visited Venado Caves might have been exposed to *H. capsulatum*, and exposure among visitors might be ongoing. In addition, histoplasmosis diagnoses are often delayed (*7*). Treatment guidelines only recommend antifungal agents for more severe cases of disease (e.g., itraconazole unless the patient is pregnant, in which case liposomal amphotericin B is recommended when treatment is indicated) (*2*,*3*). Antibiotic (i.e., antibacterial) medications are ineffective, and corticosteroids might worsen fungal infections; prescription of these medications to certain affected family members suggests that fungal infections were not initially considered as the cause of the illness. Clinicians should consider fungal illness in the differential diagnosis of patients with constitutional or pulmonary signs or symptoms after recent caving or other activities associated with risk for histoplasmosis (*8**–**10*) and should report suspected cases of histoplasmosis to local health departments as applicable (*5*).

## Footnotes

* 45 C.F.R. part 46, 21 C.F.R. part 56; 42 U.S.C. Sect. 241(d); 5 U.S.C. Sect. 552a; 44 U.S.C. Sect. 3501 et seq.

## References

[1] Lyon GM, Bravo AV, Espino A, Histoplasmosis associated with exploring a bat-inhabited cave in Costa Rica, 1998–1999. Am J Trop Med Hyg 2004;70:438–42. 10.4269/ajtmh.2004.70.438 15100461

[2] Arnold SA, Spec A, Baddley JW, IDSA 2025 guideline update on the treatment of asymptomatic histoplasma pulmonary nodules (histoplasmomas) and mild or moderate acute pulmonary histoplasmosis in adults, children, and pregnant people. Arlington, VA: Infectious Diseases Society of America; 2025. https://www.idsociety.org/practice-guideline/histoplasmosis-2025/10.1093/cid/ciaf256 40667709

[3] Wheat LJ, Freifeld AG, Kleiman MB, Infectious Diseases Society of America. Clinical practice guidelines for the management of patients with histoplasmosis: 2007 update by the Infectious Diseases Society of America. Clin Infect Dis 2007;45:807–25. 10.1086/521259 17806045

[4] Hage CA, Ribes JA, Wengenack NL, A multicenter evaluation of tests for diagnosis of histoplasmosis. Clin Infect Dis 2011; 53:448–54. 10.1093/cid/cir435 21810734

[5] Council of State and Territorial Epidemiologists. Standardized surveillance case definition for histoplasmosis. Atlanta, GA: Council of State and Territorial Epidemiologists; 2017. https://cdn.ymaws.com/www.cste.org/resource/resmgr/2016PS/16_ID_02.pdf

[6] US Embassy in Costa Rica. Health alert: histoplasmosis risks from caving activities. San José, Puerto Rico: US Embassy in Costa Rica; March 18, 2025. https://cr.usembassy.gov/health-alert-histoplasmosis-risks-from-caving-activities-march-18-2025/

[7] Benedict K, McCracken S, Signs K, Enhanced surveillance for histoplasmosis—9 states, 2018–2019. Open Forum Inf Dis 2020;9;ofaa343. 10.1093/ofid/ofaa343 32964064 PMC7491707

[8] Smith DJ, Free RJ, Thompson GR 3rd, ; Endemic Mycoses Diagnostic Algorithm Subject Matter Expert Group. Clinical testing guidance for coccidioidomycosis, histoplasmosis, and blastomycosis in patients with community acquired pneumonia for primary and urgent care providers. Clin Infect Dis 2024;78:1559–63. 10.1093/cid/ciad619 37802909

[9] CDC. Testing algorithm for histoplasmosis. Atlanta, GA: US Department of Health and Human Services, CDC; 2024. https://www.cdc.gov/histoplasmosis/hcp/algorithm/index.html

[10] CDC. Reducing risk for histoplasmosis. Atlanta, GA: US Department of Health and Human Services, CDC; 2024. https://www.cdc.gov/histoplasmosis/prevention/index.html

